# Association between inflammatory bowel disease and frailty: a two-sample Mendelian randomization study

**DOI:** 10.1007/s40520-023-02688-1

**Published:** 2024-02-06

**Authors:** Jingyi Feng, Xi Chen, Wenjing Cai, Xueying Zhou, Xuefang Zhang

**Affiliations:** 1 School of Nursing, Nanjing University of Chinese Medicine, Nanjing, Jiangsu China; 2 Hospital for Skin Diseases, Institute of Dermatology Chinese Academy of Medical Sciences, Peking Union Medical College, Nanjing, China; 3 Nanjing Hospital of Chinese Medicine Affiliated to Nanjing University of Chinese Medicine, Nanjing, 210022 China

**Keywords:** Genome-wide association studies, Inflammatory bowel disease, Causal relationship, Frailty, Mendelian randomization

## Abstract

**Background:**

An association has been identified between inflammatory bowel disease (IBD) and frailty; however, the causal nature of this connection remains uncertain. We consequently conducted a two-sample Mendelian randomization (MR) analysis to explore this particular association.

**Methods:**

We acquired distinct datasets for inflammatory bowel disease (IBD), Crohn's disease (CD), ulcerative colitis (UC), and frailty from the published genome-wide association studies (GWAS) database, meticulously selecting instrumental variables (IVs). Subsequently, we employed a bidirection MR to examine the causal relationship between IBD (including CD and UC) and frailty. We utilized statistical methods, with a primary emphasis on inverse-variance weighted (IVW), accompanied by a series of sensitivity analyses to confirm heterogeneity and pleiotropy influenced the outcomes of the MR.

**Results:**

We found positive causal effects of genetically increased frailty risk on IBD (OR: 1.015, 95% CI 1.005–1.025, *P* = 0.004). Furthermore, when scrutinizing specific IBD subtypes, both Crohn's disease (CD) and ulcerative colitis (UC) demonstrated an increased predisposition to frailty (OR: 1.018, 95% CI 1.01–1.027, *P* < 0.05) and (OR = 1.016, 95% CI 1.005–1.027, *P* < 0.05). Nevertheless, despite the consistent trends observed in the weighted median and MR-Egger regression analyses for both conditions, statistical significance remained elusive. Notably, the results of the inverse MR analysis did not establish an association between frailty and an elevated risk of IBD development.

**Conclusions:**

Our research indicates that IBD, encompassing both CD and UC, may augment the propensity for frailty. Clinical practitioners must prioritize early frailty assessment in individuals afflicted with inflammatory bowel disease, inclusive of Crohn's disease and ulcerative colitis, facilitating proactive measures and timely interventions. However, our findings do not provide evidence supporting a causal effect of frailty on IBD (including CD and UC). Consequently, further studies are essential to explore the intricate mechanisms that clarify the effect of frailty on IBD.

**Supplementary Information:**

The online version contains supplementary material available at 10.1007/s40520-023-02688-1.

## Introduction

Inflammatory bowel disease (IBD), encompassing two major forms, crohn's disease (CD) and ulcerative colitis (UC), is characterized as a chronic, immune-mediated, non-specific inflammatory disorder [1]. Currently, the incidence and prevalence of IBD are globally increasing and have exceeded 0.3% [1]. Symptoms of IBD frequently manifest as diarrhea, abdominal pain, blood in the stool, weight loss, malaise, and fever [2]. These symptoms can result in unfavorable consequences such as strictures, fistulas, surgical procedures, and disability [3], all of which substantially impact the quality of life for affected individuals. Additionally, IBD compounds the healthcare system and socio-economic burden due to its lifelong nature without a cure [4, 5].

With the ongoing global aging trend, the incidence and prevalence of elderly patients with IBD are on the rise, and it is anticipated that by 2030, over one-third of IBD patients will be aged over 60 years [6]. Frailty, characterized by diminished physiologic reserves across multiple systems and decreased resistance to stress [7], exhibits a prevalence exceeding 65% among the elderly population [8]. Symptoms include weight loss, decreased grip strength, reduced physical activity, fatigue, and slowed gait speed [9].

These symptoms overlap with common manifestations of IBD. Kochar et al. [10] have posited a correlation between IBD and frailty, citing common underlying mechanisms and symptomatology as supporting evidence. In a recent nationwide cohort study, it was determined that the overall prevalence of frailty, encompassing both low and high-risk categories, among individuals diagnosed with IBD was 61% [5]. This study also highlighted that individuals with IBD had a heightened vulnerability to developing frailty when compared to the general population [5]. Furthermore, numerous investigations have consistently emphasized the strong association between frailty and adverse outcomes in IBD patients, including increased mortality, hospitalization rates, and the risk of readmission [6, 11].

Drawing from the findings of our present investigation, we hypothesize a substantial connection between IBD and frailty. Nevertheless, the predominant focus of existing research has centered on assessing frailty’s impact on adverse clinical outcomes in patients with IBD. These studies have often overlooked elucidating the intricate relationship between IBD and frailty, and they have generally omitted in-depth exploration of the potential causal links between these two entities. It is important to acknowledge that observational studies, which constitute the majority of current research, come with inherent limitations, such as susceptibility to confounding factors and the potential for reverse causality, which can influence the observed results.

Traditional approaches to establish causality typically involve the use of randomized controlled trials (RCTs). While RCTs offer a degree of control over extraneous variables and allow for the inference of causality, their implementation necessitates substantial human, material, and financial resources, and is bound by stringent ethical and moral constraints [12]. Mendelian randomization (MR) is a methodology that employs genetic variation as an instrumental variable (IV) to ascertain causality. This approach proficiently mitigates the impact of confounding and other extraneous variables. Additionally, it leverages publicly accessible genome-wide association study (GWAS) datasets, thereby ameliorating the limitations inherent in randomized controlled trials (RCTs).

Presently, the causative association between IBD and frailty remains inconclusive. This study will employ a two-sample MR analysis approach to investigate the causal relationship between IBD and frailty, yielding significant clinical and practical insights.

## Methods and design

### Research design

We employed a two-sample MR analysis to scrutinize the causal linkage between IBD and frailty. The primary focal point of our study rested on IBD, encompassing both CD and UC.

Instrumental variable (IV) is an external factor utilized to establish a causal link between an exposure and an outcome. It represents a factor associated with the exposure, usually a hypothesized causal risk factor. Crucially, it operates independently of any confounding factors in the exposure-outcome association and solely affects the outcome through the exposure factor. Genetic variants emerge as robust candidates for IVs due to their inherent stability—they remain unchanged from the time of conception and are not influenced by environmental factors, mitigating the risk of reverse causality [13].

We meticulously selected single nucleotide polymorphisms (SNPs) with established significant associations with IBD to serve as IVs. These IVs were employed in the analysis of frailty as the outcome variable. Furthermore, we rigorously considered the possibility of reverse causality when frailty might be considered an exposure factor.

**Fig. 1:**
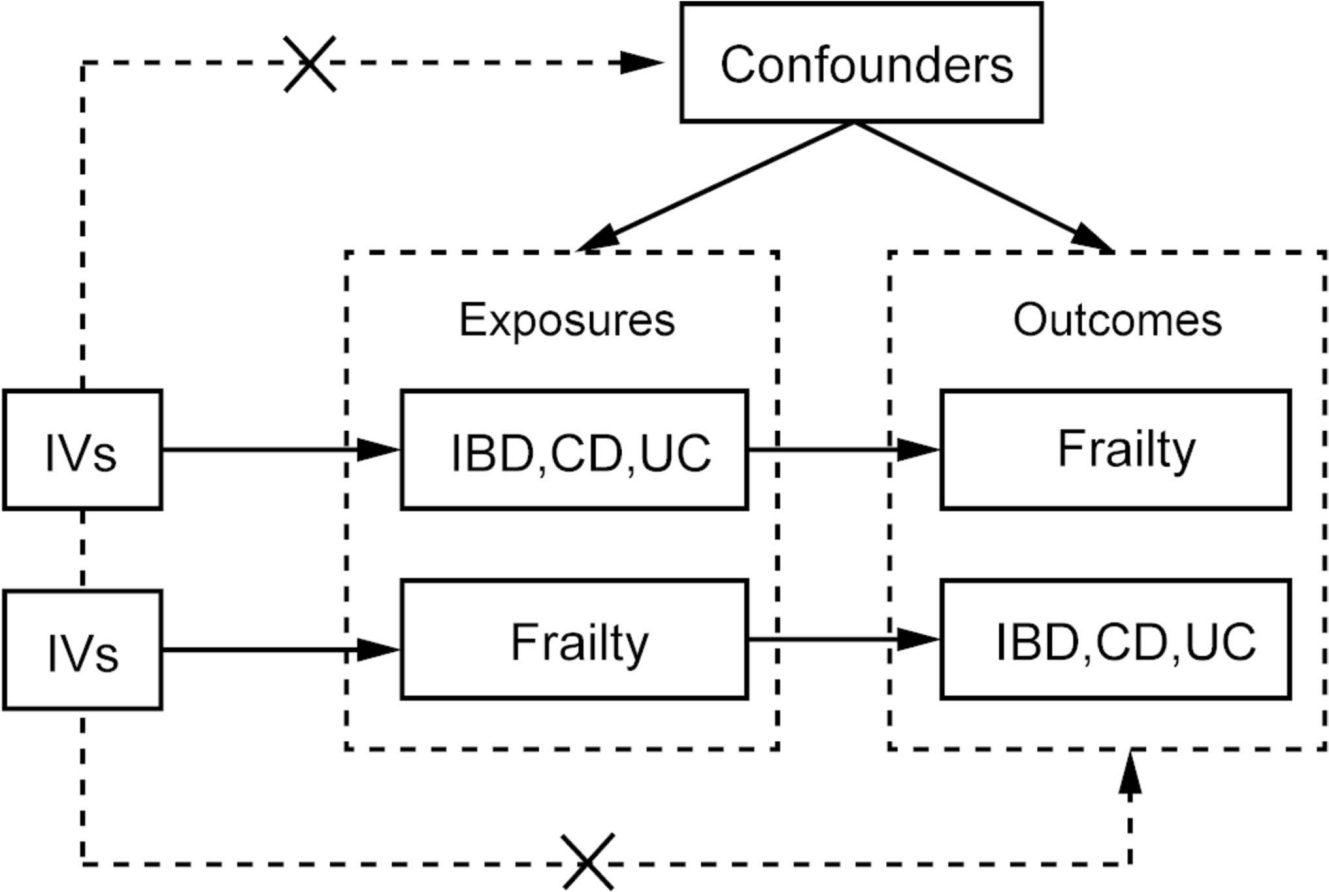
Fundamentals of Mendelian randomization (MR) studies. IVs, instrumental variables. IBD, inflammatory bowel disease; CD, Crohn's disease; UC, ulcerative colitis

The methodological framework we adopted in our study satisfies three fundamental assumptions, as illustrated in Fig. 1: (1) The IVs exhibit a close association with the exposure factors; (2) The IVs are devoid of correlations with the outcome factors, with only the exposure factors affecting the outcome; (3) The IVs remain independent of any potential confounding factors. The design of the study is displayed in Fig. 1.

## Data sources

The diagnosis of frailty in this study relied upon the utilization of the Frailty Index (FI), which is predicated on the cumulative assessment of health deficits. Genetic data pertaining to frailty were sourced from a comprehensive meta-analysis of a substantial GWAS incorporating data from the United Kingdom Biobank and the Swedish TwinGene database [14]. This dataset encompassed a total of 175,226 participants of European descent, consisting of 9,061 females and 84,607 males, with ages spanning from 41 to 93 years.

For our analysis of genetic data related to IBD, we exclusively incorporated populations of European ancestry. Our dataset comprised a total of 12,882 individuals diagnosed with IBD and 21,770 healthy controls. Within this cohort, we further classified patients into two subtypes: CD, which included 5,956 patients, and UC, which encompassed 6,968 patients. Correspondingly, each subtype was matched with their respective healthy controls, totaling 14,927 controls for CD and 20,464 for UC [15].

GWAS data on both IBD, CD, UC, and frailty are available on the GWAS catalog database (https://www.ebi.ac.uk/gwas/home).

### Selection of instrumental variables

The selection of optimal IVs from the GWAS data was conducted with meticulous scrutiny. Initially, we identified SNPs that exhibited a robust association with the exposure variable, meeting a stringent significance threshold (*P* < 5 × 10^–8^). When choosing genome-wide IVs associated with frailty, we adjusted the significance threshold (*P* < 5 × 10^–5^) to accommodate the limited number of SNPs. Subsequently, to minimize potential bias due to linkage disequilibrium (LD), we employed the “clump” program, setting the parameters with r^2^ = 0.001 and a distance of 10,000 kb. Subsequently, an extensive search was conducted in the PhenoScanner database (http://www.phenoscanner.medschl.cam.ac.uk/) to identify the confounding variables. Variables associated with grip strength, step speed, body mass index (BMI), smoking, obesity, alcohol consumption, diabetes, rheumatoid arthritis, hypothyroidism, and depression single-nucleotide polymorphisms (SNPs) were systematically eliminated. This enabled us to identify SNPs linked to confounding variables associated with the exposure outcomes, and we performed manual removal of SNPs displaying pleiotropy [16]. Additionally, we assessed the correlation of IVs exposures utilizing the F statistic [17]. The formula for the F-statistic is: [R^2^/(1 − R^2^)] × [(n − k − 1)/k]. SNPs with F values below 10 are generally considered weak IVs susceptible to bias. In our study, all F values exceeded 10. The final selection of IVs underwent the rigorous screening steps outlined above.

### Statistical analysis

In this study, we applied a comprehensive array of analytical techniques, including the inverse-variance weighted (IVW), weighted median, and MR-Egger methods, to evaluate the effect estimates pertaining to the causal association between the exposure and outcome variables. Our primary analytical method was the IVW approach, recognized for its precision and reliability. This method, as cited in reference [18], computes a weighted mean of individual rate estimates derived from all IVs. Additionally, we utilized the weighted median method as well as the MR-Egger method as auxiliary analytical tools. The weighted median estimate, akin to the IVW method, demonstrates high efficiency when at least 50% of the weights originate from valid IVs [19]. The MR-Egger method, capable of weighted linear regression of gene outcome coefficients, was employed, though it is less efficient than IVW and susceptible to the influence of weak IVs [19]. The MR outcomes were presented in terms of odds ratios (OR) along with their corresponding 95% confidence intervals (CI).

We performed a sensitivity analysis of the results of this study to test the validity and robustness of the IVW results. We used the Cochran Q statistic to characterize the heterogeneity, which was displayed using a funnel plot [20]. When there was heterogeneity in the results, the random effects model of IVW was used, otherwise, the fixed effects model was used. We used MR-Egger regression to assess the presence of horizontal pleiotropy, which was indicated if it showed a significant intercept (*P* < 0.05) [21, 22]. We also used the MR-PRESSO method to detect outliers and remove abnormal SNPs to estimate corrections [23]. In addition to this, we performed a leave-one-out analysis to exclude a SNP from having a disproportionate effect on the estimates [23].

All statistical analyses were conducted using the “TwoSampleMR” package within the R 4.2.3 statistical software.

## Results

**Fig. 2:**
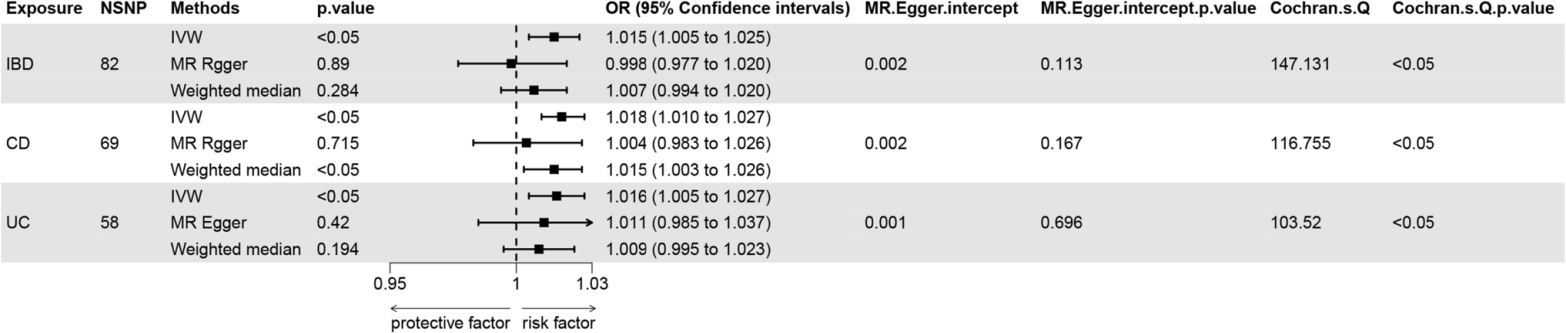
MR analysis results of IBD, CD and UC with frailty

To investigate whether there is an association between IBD and frailty, we performed an MR analysis. The results showed that IBD, CD, and UC all had a significant effect on frailty (Fig. 2). The pertinent SNP information following the screening process is provided in Supplementary 1.

### IBD

**Fig. 3:**
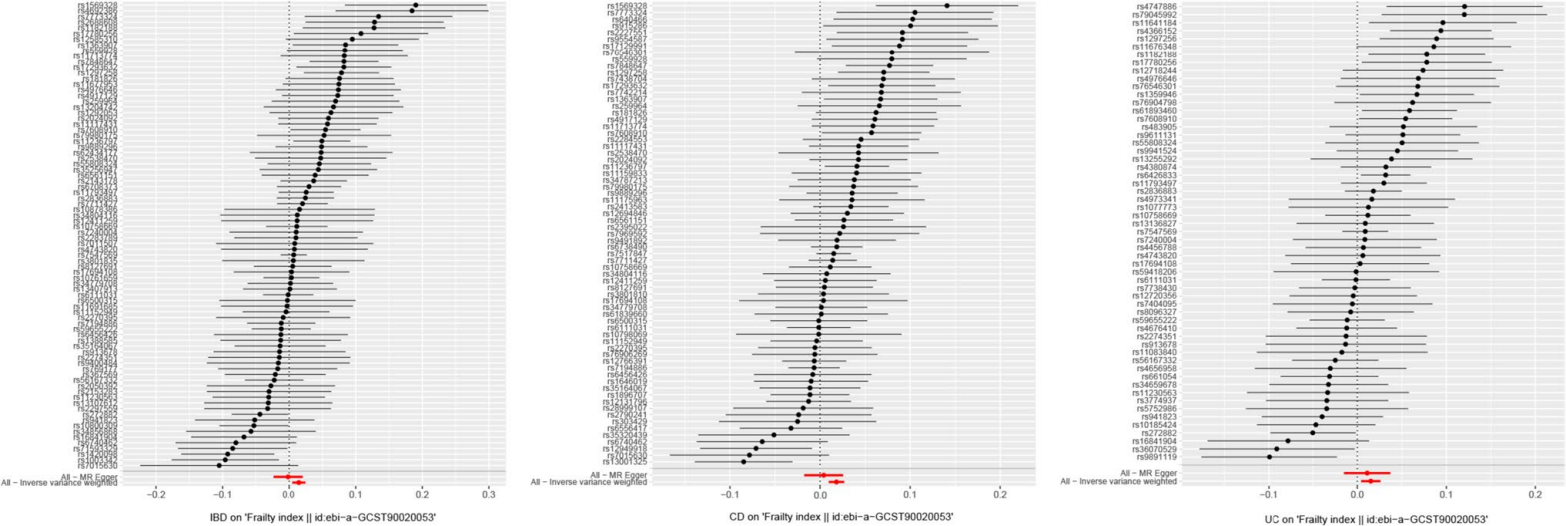
The causal effect of exposure on the outcome for each SNP. From left to right: IBD, CD, UC

**Fig. 4:**
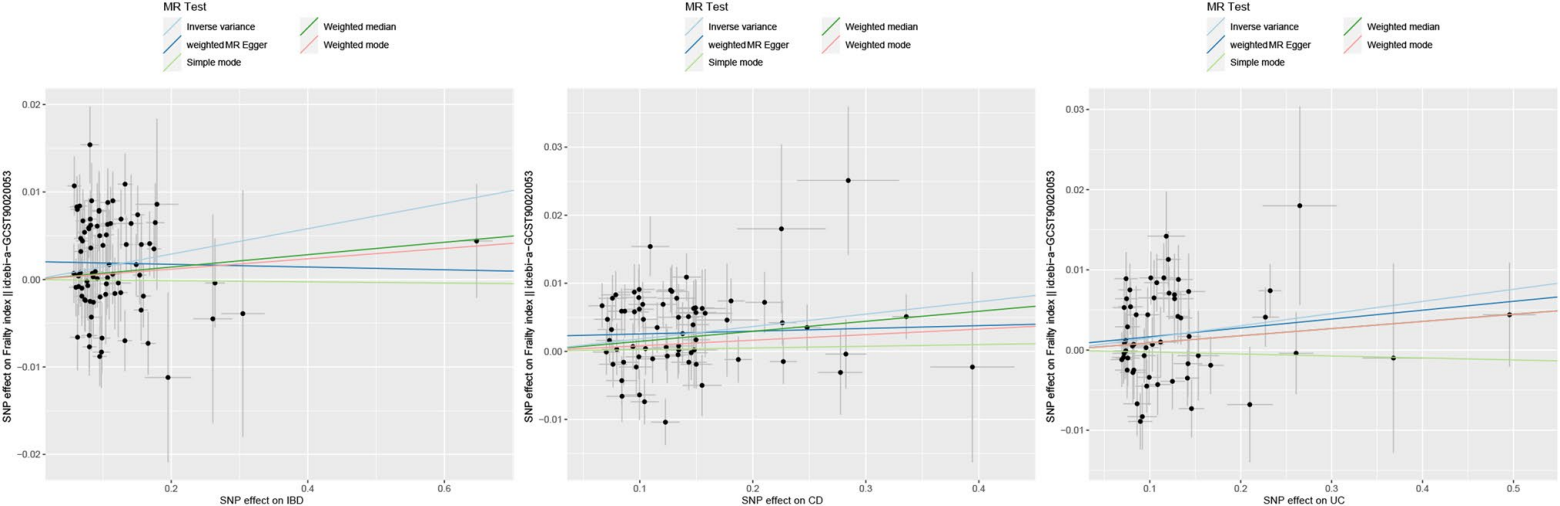
Scatter plots of causal estimates from genetically predicted IBD on frailty. From left to right: IBD, CD, UC

**Fig. 5:**
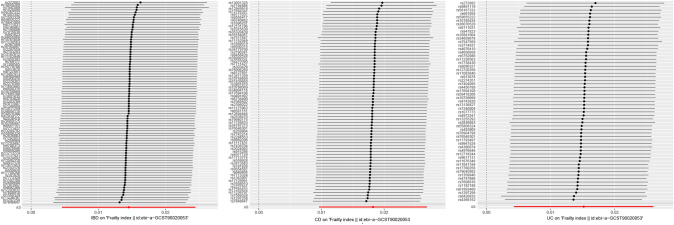
The leave-one-out sensitivity analysis for each. From left to right: IBD, CD, UC

Following a meticulous screening process for IBD and the removal of outliers through preliminary sensitivity analyses, a total of 82 SNPs. Detailed information pertaining to these SNPs can be found in the Appendix. Subsequently, these IVs underwent assessment for potential weakness, with F-statistics ranging from 53.371 to 1907.816, all surpassing the threshold of 10, indicating the absence of weak IVs. The outcomes consistently demonstrated a coherent direction of effect across various analytical methods, including the IVW, WM, Simple Mode, and Weighted Mode approaches. Specifically, in the sensitivity analysis employing the IVW method, a positive association between IBD and frailty was observed (OR: 1.015, 95% CI 1.005–1.025, *P* = 0.004). Conversely, the weighted median method did not reveal a statistically significant association between IBD and frailty (*P* = 0.284). To further ascertain the robustness of the causal associations, this study conducted a heterogeneity test and assessed horizontal gene pleiotropy. The MR-Egger regression revealed no evidence of pleiotropy (*P* = 0.113) but did indicate heterogeneity (Q: 147.131, *P* < 0.05). Utilizing the Leave-one-out method, it was evident that the removal of any one of the remaining 82 SNPs did not alter the results, reinforcing the study’s robustness (Figs. 2, 3, 4, 5).

### CD

In the case of CD, following a comprehensive screening process and the removal of outliers, a total of 69 SNPs were identified and retained as IVs, with 35 of them exhibiting associations with IBD. The sensitivity analysis, employing the IVW method, consistently indicated a positive effect of CD on frailty (OR: 1.018, 95% CI 1.01–1.027, *P* < 0.05). Similarly, the WM method yielded results in support of this conclusion (OR: 1.015, 95% CI 1.003–1.026, *P* < 0.05). Conducting the MR-Egger method, we observed no evidence of pleiotropy (*P* = 0.167), although heterogeneity was apparent (Q: 116.755, *P* < 0.05). Furthermore, employing the Leave-one-out method, it was evident that the remaining 69 SNPs consistently aligned to the right of the null line, reinforcing the study's stability (Figs. 2, 3, 4, 5).

### UC

In the context of UC, a meticulous screening process was undertaken, and four outliers were removed, resulting in the inclusion of a total of 58 SNPs as IVs, with 22 of them exhibiting associations with IBD. Remarkably, all F-statistics, ranging from 63.199 to 866.259, exceeded the threshold of 10, affirming the absence of weak IVs. Employing the IVW method, our analysis indicated a positive effect of UC on frailty (OR = 1.016, 95% CI 1.005–1.027, *P* < 0.05). Furthermore, following assessments for heterogeneity and pleiotropy, the MR-Egger method revealed no evidence of pleiotropy (*P* = 0.696) but did indicate the presence of heterogeneity (Q: 103.520, *P* < 0.05). Conducting the Leave-one-out method, it became evident that the remaining 58 SNPs consistently aligned to the right of the null line, affirming the study's stability (Figs. 2, 3, 4, 5).

## Frailty

To further explore whether frailty affects IBD, we performed a reverse MR analysis. Our results suggest that frailty may not have a causal effect on IBD. Preliminarily, frailty does not increase the pathogenic risk of IBD (OR: 0.777, 95% CI 0.173–3.488, *P *= 0.742), including the two subtypes of IBD: CD (OR: 0.763, 95% CI 0.151–3.855, *P* = 0.744) and UC (OR: 0.620, 95% CI 0.142–2.712, *P* = 0.525) were not causally related. There was no horizontal pleiotropy suggested by MR-Egger regression for IBD (*P* = 0.912), CD (*P* = 0.901), and UC (*P* = 0.779), but there was no significant relationship between IBD (Q: 157.899,* P* = 2.029 × 10^–29^), CD (Q: 125.161; *P* = 1.170 × 10^–22^), and UC (Q: 96.173, *P* = 9.33 × 10^–17^) all showed evidence of heterogeneity. After using MR-PRESSO again to remove outliers, the conclusions drawn remained unchanged. The results of the reverse MR analysis are presented in Table 1 of the Supplementary 2.

## Discussion

This study employs a two-sample MR approach to investigate the causal relationship between IBD, encompassing both CD and UC, and the development of frailty. The results showed that IBD was associated with an increased risk of frailty (OR = 1.015, 95% CI 1.005–1.025, *P* = 0.004). For subtypes of IBD, both CD and UC were associated with an increased risk of frailty (OR: 1.018, 95% CI 1.010–1.027, *P* < 0.05) , (OR = 1.016, 95% CI 1.005–1.027, *P < *0.05). Despite observed heterogeneity in the findings, we applied the IVW random-effects model and conducted sensitivity analyses, demonstrating the overall robustness of our results. According to the MR analysis, the MR-Egger method for IBD had an opposite slope to the other methods, suggesting that the results need further validation. Therefore, we analyzed the subtypes of IBD, which resulted in consistent slopes for all five analysis methods, and the sensitivity analysis results suggested reliable results. However, inverse MR analysis indicates that there is no reverse causal relationship between IBD (including CD and UC) and frailty.

The existing body of evidence concerning the relationship between IBD, specifically CD, and UC, and frailty predominantly comprises observational studies, with fewer investigations of alternative study designs. Prior investigations have established frailty as a significant risk factor associated with IBD. A nationwide cohort study [5] showed that 49% of IBD patients had a low risk of frailty and 12% of IBD patients had a high risk of frailty, and that frailty was more prevalent in IBD patients than in non-IBD patients [24]. A study by Faye et al. [11] showed that the prevalence of frailty in hospitalized IBD patients rose between 2010 and 2014 from 10.2% to 11.45%. A meta-analysis by Dai et al. [25] showed higher rates of hospitalization, mortality, and infections in patients with frailty IBD compared to non-frailty IBD patients (OR: 1.66, 95% CI 1.30–2.11, OR: 2.11, 95% CI 1.15–3.88, OR: 1.73, 95% CI 1.29- 2.32). Fons et al. [26] also stated that frailty is an important predictor of clinical outcomes in IBD.

Several reasons help explain the association between IBD and frailty. First, chronic inflammatory response plays a key role in frailty and IBD. Contemporary research indicates an association between frailty and a chronic systemic inflammatory response [27]. Furthermore, frailty frequently coincides with the aging process, which is known to elicit a prolonged inflammatory response. Studies [28, 29] have shown that aging leads to elevated serum interleukin (IL)-6, C-reactive protein (CRP), and tumor necrosis factor (TNF)-αconcentrations. Whereas the pathogenesis of IBD is generally believed to be the result of immune dysregulation due to the interaction of genetic and microbial factors, this stimulus will also accelerate cellular senescence and promote increased production of inflammatory factors such as tumor necrosis factor (TNF) α, interleukin (IL)-1, IL-6, IL-18, IL-12 and IL-23 [30–32]. Secondly, sarcopenia is considered to be a pre-existing manifestation of frailty. Nardone et al. [33] summarized the reasons for the existence of a correlation between IBD and sarcopenia, including inflammation, intestinal ecological dysregulation, and malnutrition. Previous investigations [34, 35] have demonstrated an association between Interleukin-6 (IL-6) and muscular dystrophy as well as sarcopenia in the elderly population. Additionally, the Janus Kinase (JAK)/Signal Transducer and Activator of Transcription (STAT) cellular signaling pathway, initiated by the binding of IL-6 to its receptor, has been closely linked to the pathogenesis of IBD. The “leaky gut syndrome” caused by IBD also contributes to the development of IBD and sarcopenia [36]. In addition, IBD patients suffer from intestinal malabsorption, which affects skeletal muscle mass [37].

Our study offers evidence supporting an association between IBD, CD, and UC with an elevated risk of frailty, though the reverse causation is not observed. Nonetheless, the pathophysiologic mechanisms of IBD leading to frailty are not fully elucidated at present. More in-depth studies are needed in the future to further explore the mechanisms of association between IBD and frailty for clinical prevention.

The strengths of our study are that, for the first time, we assessed the causal effect of IBD on frailty using a bidirection two-sample MR approach. This approach is less susceptible to confounders and reverse causality than traditional observational studies; this method is closer to randomized assignment than randomized controlled trials; and second, we subtyped IBD to causally relate CD and UC to frailty separately, elucidating the different effects of specific diseases on the impact of frailty and avoiding the influence of two subtypes on the results.

Our study presents several noteworthy limitations. Firstly, our data selection was confined exclusively to the European population, which potentially restricts the generalizability of our findings to more diverse populations and ethnic groups. Secondly, while diligent efforts were made to mitigate the influence of IVs on the outcome, complete elimination of their impact may not have been achieved. Thirdly, the presence of heterogeneity within our study raises concerns about the overall reliability of the results. Lastly, our study utilized the Frailty Index as the diagnostic instrument for frailty. In future research endeavors, we can contemplate referencing the criteria outlined in the Fried frailty phenotype, which subdivides frailty into five distinct symptoms, thereby allowing for a comprehensive analysis of the causal relationship between IBD and each individual symptom of frailty.

## Conclusion

This study is the first to use MR to explore the causal relationship between IBD and frailty, which has clinical implications. The study suggests that IBD (including CD and UC) may increase the prevalence of frailty. Therefore, it is important for clinical staff to pay attention to early screening for frailty in the population of patients with IBD (including CD and UC), and to put in place relevant preventive, therapeutic and management measures in advance. However, our findings do not provide a causal effect of frailty on IBD (including CD and UC). Consequently, further studies are essential to explore the intricate mechanisms that clarify the effect of frailty on IBD.                                                                                                                                       

### Supplementary Information

Below is the link to the electronic supplementary material.

Supplementary file1 (XLSX 49 KB)

Supplementary file2 (DOCX 11887 KB)

## Data Availability

The data used in this article is included in the supplementary 1. GWAS data of IBD and frailty can be found in GWAS catalog database (https://www.ebi.ac.uk/gwas/home).
