# Supplementary material for: Association between inflammatory bowel disease and frailty: a two-sample Mendelian randomization study
Source: Aging Clin Exp Res. 2024 Feb 6;36(1):21. doi: 10.1007/s40520-023-02688-1 (PMC10847216; doi:10.1007/s40520-023-02688-1)
Supplement: Supplementary file 2 — Supplementary file2 (DOCX 11887 KB) [file 40520_2023_2688_MOESM2_ESM.docx]

| Exposure | Outcome | NSNP | MR-Egger | |  | IVW | |
| --- | --- | --- | --- | --- | --- | --- | --- |
|  |  |  | OR（95%*CI*） | P |  | OR（95%*CI*） | P |
| Frailty | IBD | 11 | 0.496(1.862×10^-4^～1320.520) | 0.865 |  | 0.777（0.173 to 3.488） | 0.742 |
| Frailty | CD | 11 | 0.574 (1.145×10^-4^～2876.521) | 0.901 |  | 0.763（0.151 to 3.855） | 0.744 |
| Frailty | UC | 11 | 0.320 (1.389×10^-4^～736.392) | 0.779 |  | 0.620（0.142 to 2.712） | 0.525 |

Table 1 The inverse MR analysis results of IBD, CD and UC with Frailty

**
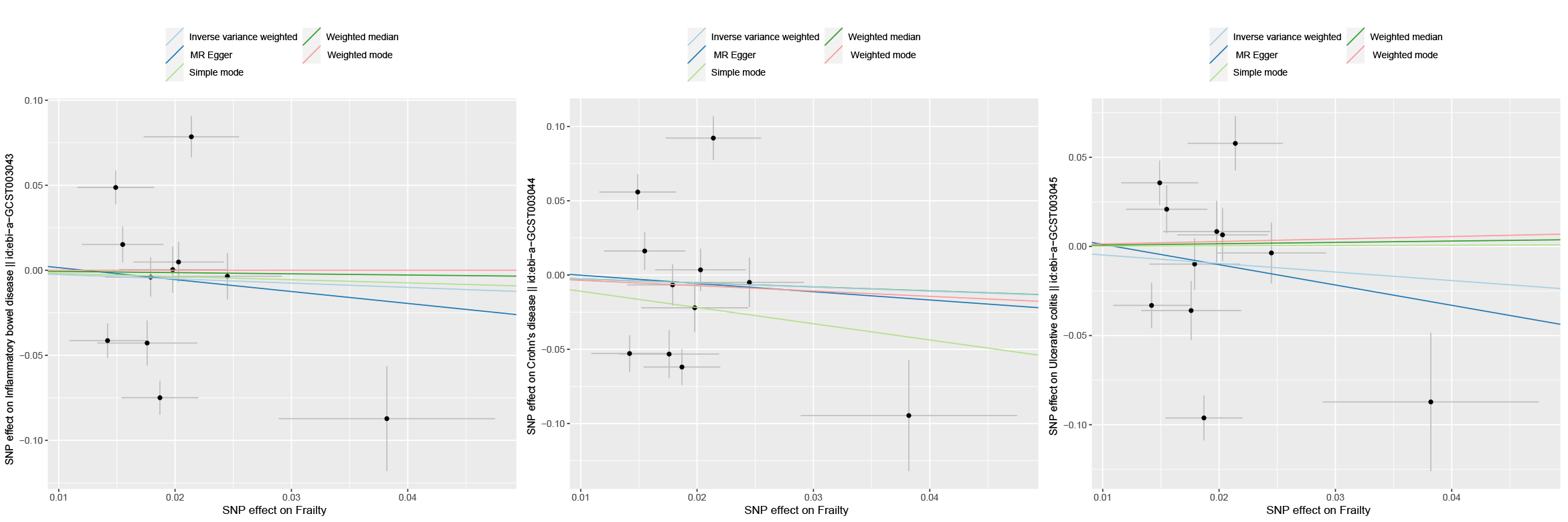
**

**Fig.6** The scatterplot of exposure-related frailty on IBD, CD, and UC


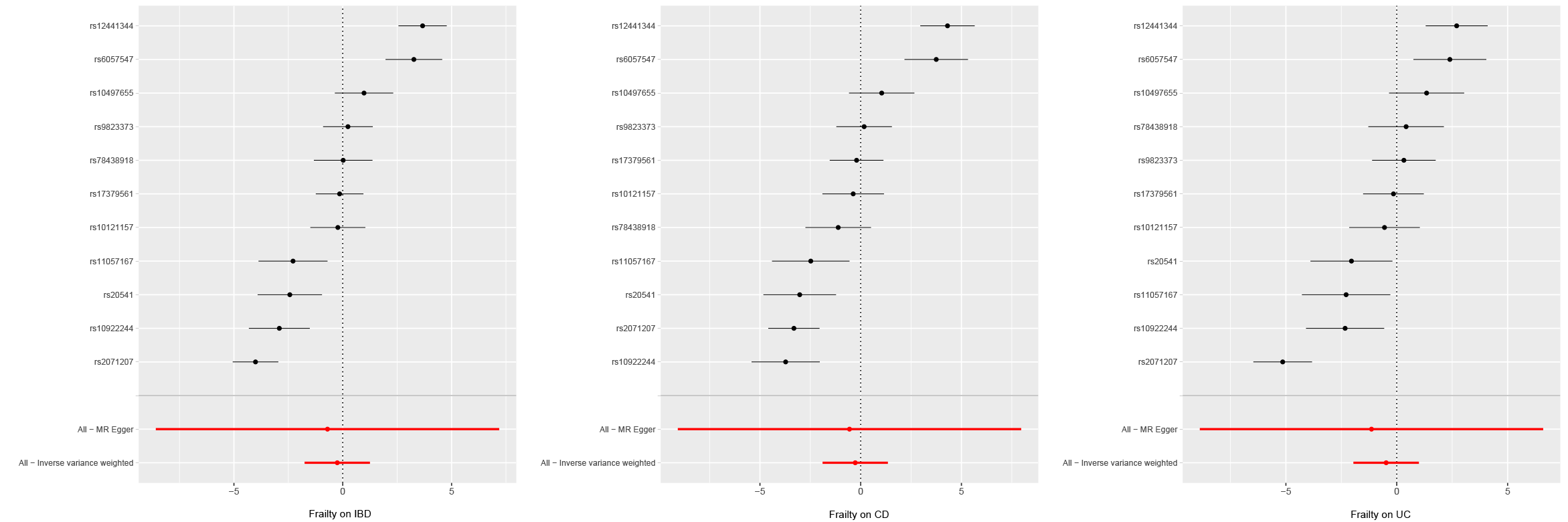


**Fig.7** The forest plots of exposure-related frailty on IBD, CD, and UC
